# Reviewers BJCVS 31.5

**Published:** 2016

The Brazilian Journal of Cardiovascular Surgery (BJCVS) is grateful for the reviewers, listed below, which collaborated in this edition. Without their work would be impossible to keep the high scientific standard of our journal.

Carlos Manuel de Almeida Brandão
Claudia Bernardi Cesarino
Eduardo Augusto Victor Rocha
Ektor Correa Vrandecic
Elaine Soraya Barbosa de Oliveira
Fábio Antônio Gaiotto
Fábio Binhara Navarro
Henrique Murad
Jarbas Jakson Dinkhuysen
José Carlos Dorsa Vieira Pontes
Luciano Albuquerque
Omar Asdrúbal Vilca Mejía
Paulo Roberto Barbosa Évora
Tomas Salerno
Valéria Papa
Vinicius José da Silva Nina

